# Biomarkers of Pesticide Exposure in a Traditional Brazilian Amazon Community

**DOI:** 10.3390/ijerph21111396

**Published:** 2024-10-23

**Authors:** Cristal M. T. Fona, Antonio M. M. Miranda, Maria I. Jesus, Viviane M. Silva, Cássia C. S. Rocha, Amilton C. G. Costa, Rosivaldo A. Mendes

**Affiliations:** 1 Postgraduate Program in Health Surveillance and Epidemiology, Evandro Chagas Institute, Ananindeua 67030-000, Brazil; cristalnutricionista@gmail.com (C.M.T.F.); vivianemonteiro95@outlook.com (V.M.S.); 2 Environment Section, Evandro Chagas Institute, Ananindeua 67030-000, Brazil; marcosmota@iec.gov.br (A.M.M.M.); mariajesus@iec.gov.br (M.I.J.); cassiarocha@iec.gov.br (C.C.S.R.); amiltoncosta@iec.gov.br (A.C.G.C.)

**Keywords:** butyrylcholinesterase, cholinesterase, cytokines, quilombola, traditional community

## Abstract

In 2008, Brazil became the country with the highest pesticide use in the world, with over one billion liters of pesticides applied to crops in 2009. The impacts of these products on public health are wide-ranging. Vast territories are affected, involving different population groups, such as workers in various fields of activity, the population that consumes contaminated food, and people living around factories, such as traditional communities. This study aimed to assess human exposure to pesticides through epidemiological and laboratory data of residents of the Santo Antônio quilombola community in Concórdia do Pará, Amazon region, Brazil. Epidemiological data were collected using a semi-structured questionnaire, which included factors such as sex, age, length of residence, and level of exposure to pesticides. The modified Ellman method was used to assess the activity of cholinesterases, and flow cytometry was performed for cytokine analysis. Analysis of collected blood samples showed that, in most cases, there was no significant reduction in the activity of acetylcholinesterase (AChE) and butyrylcholinesterase (BChE) compared to other studies in the scientific literature. Meanwhile, there was an increase in the levels of IFN-γ cytokines, especially IL-6, in all groups. The findings of this study highlight the urgent need for a comprehensive monitoring program, considering that some conditions other than pesticide exposure can alter the activities of the biomarkers used in this study.

## 1. Introduction

The use of pesticides on a global scale has become increasingly widespread, being used worldwide for pest control in agriculture, domestic purposes, and pest control related to public health [1]. In Brazil, a series of policies implemented by different governments contributed to the so-called modernization of agriculture, a process that resulted in the gradual increase in the use and commercialization of pesticides. In 2008, Brazil became a world leader in the application of pesticides, and in 2009, one billion liters of pesticides were applied to crops [2]. In 2021, Brazilian agriculture consumed around 720.870 tons of pesticides, involving 652 active ingredients from the most varied chemical groups and toxicological classifications available on the national market [3]. One of the strengths of the Brazilian economy is its agricultural production for export, with 88 million hectares cultivated in the country [4]. The primary pesticide consuming countries including China, the USA, Argentina, India, Japan, Canada, Brazil, France, Italy, and Thailand [5]. Four of the largest agricultural producers in the world are the USA, EU, China, and Brazil—together accounting for more than half of all global agricultural production value [6]. The scientific literature shows that countries such as China and the United States, among the largest consumers in the world, have published numerous studies on the impacts of these substances on the health of their populations [7]. The impact of pesticides on public health is enormous. It is implicated in fertility, induction of teratogenic and genetic defects, cancer, respiratory problems, cardiovascular, genitourinary, gastrointestinal, hematological disorders, and allergic reactions [8]. The implications affect vast territories and involve different population groups, such as workers from various fields of activity, populations that consume contaminated food, and residents close to factories and monocultures, such as traditional communities [9,10].

According to the specific chemical structure, pesticides can be classified into different classes, such as carbamates, herbicides, organochlorine, organophosphate, and pyrethroids [11]. Organophosphate and carbamate pesticides, also known as anticholinesterase pesticides, trigger the accumulation of acetylcholine in nerve endings, causing various neurobehavioral damages, triggering a series of nicotinic, muscarinic, and central nervous system effects and are referenced in cases of human poisoning [12]. Biomonitoring studies of human populations exposed to organophosphate and carbamate pesticides commonly use exposure, effect, or susceptibility biomarkers [13]. Biomarkers play an essential role, as they can suggest biological changes at an early stage, preventing health damage. Organophosphate and carbamate pesticides, which are biomarkers of exposure, can affect an individual’s health and can be assessed through biomonitoring. The effect of the biomarker can be measured by determining the activity of plasma and erythrocyte acetylcholinesterase [14]. The acute toxicity of OP is mediated by the inhibition of acetylcholinesterase (AChE), resulting in the production of nicotinic and muscarinic receptors that trigger cholinergic effects associated with central respiratory paralysis [15]. Butyrylcholinesterase (BChE), a plasma enzyme synthesized in the liver with AChE catalytic properties, is frequently used to determine acute exposure in biomonitoring programs [13]. The concentration of BChE is affected by a variety of factors, such as malnutrition, systemic inflammation and liver cell damage [16]. The specific association of BChE with Alzheimer’s disease (AD) pathology suggests these enzymes are promising targets to facilitate diagnosis and treatment monitoring of AD [17].

Pesticides have neurotoxic activity and affect the immune system and cytokine production [18]. Organophosphate pesticides stimulate a variety of immunological phenomena relevant to autism. In the CNS, acute and chronic exposure to organophosphate pesticides results in increased production of inflammatory cytokines, including IL-1Β and IL-6 in brain regions, similar to findings in the autism brain [19]. Long-lasting neurological, behavioral, and cognitive changes may occur due to excitotoxic damage to the central nervous system [20].

Quilombolas communities are ethnic groups predominantly made up of the rural or urban black population, brought to Brazil and enslaved between the 16th and 19th centuries may be potentially exposed to pesticides due to spray dispersion during application and volatilization of pesticides after their application through routes such as inhalation, dermal exposure, or ingestion [21]. They live in isolated communities that define themselves based on relationships with the land, kinship, territory, ancestry, traditions, and cultural practices. It is estimated that throughout Brazil, there are more than three thousand quilombola communities [22,23]. A group of individuals who live, work, or attend school near agricultural activities that use pesticides and whose presence is not related to work involving pesticides but whose position may lead to exposure may be associated.

Oil palm plantations are predominantly located close to quilombola communities and are crossed by watercourses, streams, rivers, and lakes. The result is the configuration of oil palm plantations as areas of environmental risk due to the possible contamination of water resources, compromising the health of the traditional communities in the surrounding area that use this water [24].

This work aims to understand the degree of environmental exposure in quilombola communities adjacent to large monocultures, which is essential for better planning public policies to protect this group. In this context, we aimed to identify possible human pesticide exposure through epidemiological and laboratory data in residents of the Santo Antônio quilombola community in Concórdia do Pará, Amazon region, Brazil.

## 2. Materials and Methods

### 2.1. Study Area

**Figure 1:**
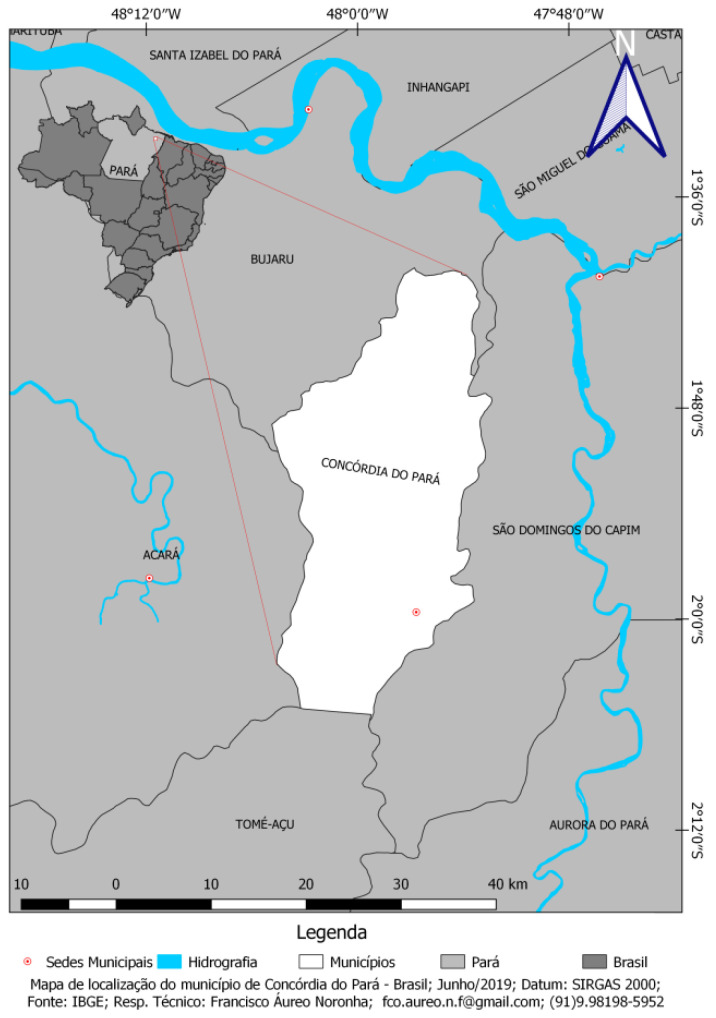
Map of the localization of the city of Concórdia do Pará—Amazon region—Brazil.

The Santo Antônio quilombola community, a unique cultural entity, was certified by the Palmares Cultural Foundation as “Quilombo Remnant” in 2008. Located in the municipality of Concórdia do Pará, in the northeast of the state of Pará (Figure 1), this municipality is close to the municipalities Acará, Mãe do Rio, and São Domingos do Capim. The total population is estimated at 33,318 inhabitants, according to projections by the Brazilian Institute of Geography and Statistics (IBGE). It has an area of 690.947 km^2^, generating a demographic density of 40.84 inhabitants/km^2^ [25]. The main economic activity is black pepper farming and subsistence agriculture, with cassava flour as the main product. Most palm oil companies in the state are concentrated in the northeast of Pará. The community participating in the study is located within the limits of the oil palm monoculture territory, with the closest plantation less than 2 km away. In 2020, the state of Pará was responsible for 98.47% of palm oil production in Brazil, reaching approximately 200 thousand hectares of harvested area [26]. The most used pesticides in palm oil cultivation are herbicides such as glyphosate, diuron, and paraquat and insecticides such as acephate, carbofuran, terbufós, chlorpyrifos, malathion, cypermethrin, diazinon, and profenofos [27].

### 2.2. Subjects and Sample Collection

This was a cross-sectional study with the participation of 188 individuals of both sexes, aged between 1 and 88 years, from the Santo Antônio community. Participants with less than one year of residence in the community were excluded from the study. Epidemiological data were collected by applying a semi-structured questionnaire to the participants. Children under 12 years of age were assisted by their parents or guardians. The socioeconomic variables selected to compose the work were gender, age, and length of residence. Whether workers reported working directly or not with pesticides was used to inform on occupational exposure.

Five milliliters of whole blood were collected from the participants, with local sanitization before the 15-min orientation of the patient before venous injection. They were transported into tubes containing 10% EDTA at 4 °C. Whole blood and serum samples were separated and frozen at −20 °C for quantification of cytokine serum in the Human Biology Laboratory and acetylcholinesterase enzyme activity in the Toxicology Laboratory, both in the Evandro Chagas Institute.

### 2.3. Analysis of Acetylcholinesterase Enzyme Activity

AChE and BChE activities were analyzed according to the method described by Ellman et al. [28], with some modifications. 20 μL of whole blood were placed in Falcon tubes with 20 mL of buffer solution d and 5,5-dithiobis-2-nitrobenzoic acid (DTNB). A total of 4 mL of this mixture was then placed in tubes identified as “whole blood” and all white followed by codes from the sample are used. Fifty µLof Salici broadest of eserine was added to the “total white” tube and held for a pre-incubation of 5 min at 30 °C. 1 mL of iodide propiothiocholine was added to all tubes and then incubated for 10 min at 30 °C. Next, 50 µL of eserine salicylate was placed in the whole blood tubes. An absorbance reading was performed on the entire blood using a UV Cary model (Varian) spectrophotometer at 420 nm.

### 2.4. Cytokine Analysis

Cytokine analysis was performed using the BD Cytometric Bead Array (CBA) Human Th1/Th2 Cytokine Kit II. Six populations of beads with different fluorescence intensities were conjugated to a specific capture antibody for each cytokine. These bead populations were visualized according to their respective fluorescence intensities from less bright to brighter. In CBA, cytokine capture beads are mixed with the fluorochrome-conjugated detection antibody and then incubated with the samples. The acquisition tubes were prepared with a 50 µL sample, 50 µL mixture of beads, and 50 µL of detection reagent. The same procedure was used to obtain a standard curve. The tubes were homogenized and incubated for three hours at room temperature in the dark. The readings were held in the unit cytometer flow, and results were generated as graphs and tables using FCAP Array^TM^ Software version 3.0. The control used in the flow cytometer was the calibration of the equipment, and its channels/filters were adjusted as recommended by the manufacturer.

### 2.5. Ethical Considerations

The study was approved by the Ethics Committee for Research with Human Beings (CEP) of the Instituto Evandro Chagas under Opinion No. 2.658.533. Informed consent was obtained from all participants, complying with the Guidelines and Regulatory Norms for Research involving Human Beings (CNS Resolution No. 466/12), using the Free and Informed Consent Term and the Free and Informed Assent Term among study participants. All data were kept confidential, with the results reported individually to the participants, respecting their privacy and rights.

### 2.6. Statistical Analysis

The information obtained by applying a specific questionnaire was stored in a spreadsheet-like database using MS Excel 7.0. Statistical tests were performed using the EpiInfo version 7.2.2.6. Mann–Whitney nonparametric tests were performed for dichotomous variables, and the Kruskal–Wallis test was performed for multivariate variables.

## 3. Results

Demographic data were divided into four groups and are described in Table 1. Of the 188 research participants, the occupationally exposed group included 25 agricultural workers (13.3%). According to the in-person questionnaire reported by these agricultural workers, all reported that they used personal protective equipment (masks, gloves, coveralls, and goggles) during pesticide application, while the remainder were environmentally exposed individuals with residences close to agricultural monoculture. The predominant age range was 20 to 59 years (56.4%), the majority were male (53.7%), and 84.0% reported living in the community for over ten years. According to the BMI classification by age group from 1 to 5 years and from 6 to 19 years, respectively, eutrophy predominated (69.2% and 90.7%), while among those aged 20 to 59 years and over 60 years, overweight prevailed (42.5% and 50.0%)

### 3.1. Cholinesterase Enzyme Activity

Contact with cholinesterase-inhibiting pesticides, such as organophosphates and carbamates, increases the risk of exposure. These substances’ primary mechanism of action is the inhibition of the cholinergic enzymes acetylcholinesterase (AChE) and butyrylcholinesterase (BChE). This causes the accumulation of the neurotransmitter acetylcholine in the synaptic cleft and induces excessive stimulation of nicotinic and muscarinic receptors in the central and peripheral nervous systems, leading to paralysis of cholinergic synaptic transmission [20].

Table 2 shows the statistical analysis of the enzyme activity of AChE and BChE according to socioeconomic characteristics. The mean and median levels of AChE are lower in the age group from 1 to 11 years old (4.89 and 4.47 UI/mL), among female participants (5.35 and 4.74 UI/mL), people who had lived in the community for between 2 and 4 years (5.27 and 3.82 UI/mL), and among workers who reported not having direct contact with pesticides (5.57 and 5.10 UI/mL).

AChE dosage according to food frequency showed lower mean and median levels among those who claimed not to consume the game (5.66 UI/mL and 5.0 1 UI/mL). The lowest average fish and seafood consumption was among those who drank it between 1 and 2 times a week (5.17 UI/mL), and the lowest median was found among those who said they did not consume them (4.46 UI/mL). The frequency of fruit consumption had a lower mean and median among those who reported eating them 1–2 times a week (5.35 UI/mL and 4.51 UI/mL). The mean and median vegetable consumption were minor among those who said they did not consume such foods (4.96 UI/3.7 mL and 3 UI/mL). There was no significant difference between the mean levels of AChE enzyme activity according to socioeconomic characteristics and food frequency.

The average and median enzyme activity of BChE by age is lower among children aged 1–11 years (4.21–3.82 UI/mL). Male and female participants had the same mean (4.64 UI/mL); however, the median was lower in males (4.54 UI/mL). The mean and median by length of residence in the community are 2–4 years (4.15 UI/mL and 3.88 UI/mL). The mean and median were lower among the participants who reported not working directly with pesticides (4.60 UI/mL and 4.54 UI/mL). There was no significant difference in the mean levels of BChE enzyme activity, according to socioeconomic characteristics.

BChE dosage according to food frequency showed lower mean and median levels among those who claimed to consume games 2–4 times a week (4.56 UI/mL and 4.36 UI/mL). The mean and median among those who consumed fish and shellfish between 5 and 6 times a week were 3.21 UI/mL and 2.91 UI/mL). Statistical significance was observed for the mean levels of BChE by the frequency of fish and shellfish consumption (*p* < 0.03). The frequency of fruit consumption had a lower mean and median among those who reported eating them 5–6 times during the week (4.08 UI/mL and 3.61 UI/mL). The mean and median were lower still among those who claimed not to consume fruit and vegetables (2.26 UI/mL and 2.26 UI/mL).

BChE levels according to BMI by age group in children aged 1 to 5 years had a lower mean and median among those at risk for overweight (3.76 Ul/mL and 3.76 Ul/mL). Regarding the 6 to 19-year-old group, the mean and median are lower among eutrophic participants (4.37 Ul/mL and 4.22 Ul/mL). Participants aged 20 to 59 showed lower mean and median among eutrophics (4.21 Ul/mL and 4.00 Ul/mL). Participants older than 60 had a lower mean among those underweight (4.43 Ul/mL), and a lower median among those who were overweight (4.42 Ul/mL). There was no significant difference between the dosage of BChE enzyme activity according to BMI classifications by age group.

### 3.2. Cytokines

Pesticides can interact with normal human biochemical processes such as immune system homeostasis. Recent studies reported that exposure to pesticides can impair immunity, reducing antimicrobial activity and disturbing the endocrine system, altering the production of inflammatory mediators such as cytokines [29]. Cytokines influence immune cell activity, differentiation, proliferation, and survival and regulate the production and activity of other cytokines. These can either enhance (pro-inflammatory) or attenuate (anti-inflammatory) the inflammatory response. Cytokines can also regulate innate or adaptive immunity, hematopoiesis, inflammatory processes, and many other important activities for the human body. Consequently, the levels of cytokines are recognized as an essential indicator for evaluating clinical disorders [30].

Table 3 presents the statistical analysis of the dosage of cytokines (IFN-γ, TNF, IL2, IL4, IL6, and IL10), according to socioeconomic characteristics and food frequency. The mean level of IFN-γ was higher among the group over 60 years old (9.28 pg/mL). Female participants had a higher mean than males (9.35 pg/mL); this group showed statistical significance between the mean levels of IFN-γ (*p* < 0.02). People living in the community for between 5 and 10 years had a higher mean (10.21 pg/mL). Participants who reported not having direct contact with pesticides had a higher mean (9.03 pg/mL), and statistical significance was observed between the mean levels of IFN-γ in this group (*p* < 0.04).

The dosage of IFN-γ according to food frequency showed a lower mean and median level among those who claimed not to consume game (8.75 pg/mL and 8.92 pg/mL), and a higher mean level among those who drank it 3–4 times during the week (10.89 pg/mL). The average and median were lower among those who consumed fish and seafood between 1 and 2 times a week (8.63 pg/mL and 8.94 pg/mL, respectively), and the average was higher among those who said they did not consume these foods (10.99 pg/mL). The mean and median were lower among those who reported eating fruit 1–2 times during the week (8.39 pg/mL and 8.38 pg/mL), and the mean was higher among those who ingested fruit 5–6 times a week (9.29 pg/mL). The lowest average was among those who claimed not to consume vegetables (8.51 pg/mL) compared to those who did. The lowest median was among those who drank between 1 and 2 times a week (8.56 pg/mL), and the mean was higher among non-responders (13.23 pg/mL). However, the thoroughness of our research is evident in the lack of statistically significant differences between the mean IFN-γ levels according to food frequency. There was no significant difference in the TNF dosage due to socioeconomic characteristics.

There was a statistically significant difference between the average levels of TNF, which is determined according to the frequency of fish and shellfish consumption (*p* > 0.001), and by those who did not consume fruit (*p* > 0.0003). The mean and median were lower among those who the fruit daily (8.20 pg/mL and 8.13 pg/mL, respectively), and the mean was higher among those who said they did not consume them (9.63 pg/mL). The mean and median were lower among those who did not respond (7.91 pg/mL and 7.91 pg/mL, respectively), and the mean was higher among those who consumed fruit 1–2 times a week (9.21 pg/mL).

The mean and median levels of IL-2 were lower in those between 1 and 11 years old (9.18 pg/mL and 9.10 pg/mL). The mean was higher among young people aged 12–19 (9.61 pg/mL). Female participants had the lowest mean and median (9.38 pg/mL and 9.18 pg/mL), people living in the community between 2 and 4 years old had the lowest mean and median (8.84 pg/mL and 8.43 pg/mL), and those who lived for ten years had the highest average (9.53 pg/mL). Workers with direct contact and those who did not have direct contact with pesticides had identical means (9.45 pg/mL), while the median was lower among those who did not have direct contact with pesticides (9.24 pg/mL). There was no significant difference in the TNF dosage due to socioeconomic characteristics.

There was a statistically significant difference between the mean levels of IL-2 according to the frequency of fruit consumption (*p* > 0.0003). The mean and median were lower among those who did not respond (8.43 pg/mL and 8.43 pg/mL, respectively), and the mean was higher among those who consumed 3–4 times a week (9.74 pg/mL).

There was no significant difference in the IL-4 dosage due to socioeconomic characteristics.

The dosage of IL-4 according to food frequency showed lower mean and median levels among those who stated they did not consume wild animals (9.78 pg/mL). The median was lower, while the mean level was higher among those who consumed wild animals 3 to 4 times during the week (8.99 pg/mL and 11.16 pg/mL). The mean and median were lower among those who consumed fish and seafood 1–2 times a week (9.68 pg/mL and 9.27 pg/mL). There was no significant difference in the mean levels of IL-4 according to food frequency.

The mean level of IL-6 was lower among young people aged 12–19 years (17.85 pg/mL), while the lower median and the highest mean were found among those older than 60 (12.91 pg/mL and 35.92 pg/mL). Male participants had lower mean and median levels (21.39 pg/mL and 13.34 pg/mL). Statistical significance was observed between the mean levels of this cytokine by sex (*p* < 0.008). People living in the community for between 5 and 10 years had a lower mean (19.04 pg/mL), while the lowest median and the highest mean were found among those living for more than 10 years (13.67 pg/mL and 22.85 pg/mL). Workers who reported having direct contact with pesticides had lower mean and median values (17.11 pg/mL and 13.78 pg/mL).

As for IL-6 levels according to BMI by age group, among children aged 1 to 5 years, the mean and median levels are lower among eutrophic children (17.11 pg/mL and 14.50 pg/mL), the mean is higher among those classified at risk for overweight (35.43 pg/mL). Regarding the 6 to 19-year-old group, the mean and median are lower among eutrophic participants (17.57 pg/mL and 13.00 pg/mL), while the mean is higher among overweight participants (18, 18 pg/mL). Participants aged 20 to 59 years showed a lower mean among participants classified as obese (16.79 pg/mL), a lower median among eutrophic individuals (13.30 pg/mL), and the highest mean among those classified as overweight (25.72 pg/mL). Participants older than sixty years had a lower mean and median among those classified as underweight (13.09 pg/mL and 12.78 pg/mL), whereas the highest mean is among eutrophics (68.09 pg/mL); there was statistical significance regarding IL-6 dosage by BMI classification in the elderly (*p* < 0.04). There was no significant difference in the mean levels of IL-6 according to food intake frequency.

The mean and median levels of IL-10 were lower among participants aged between 1 and 11 years (9.28 pg/mL and 9.33 pg/mL). In contrast, the highest mean was found among adults aged 20–59 years (9.84 pg/mL). Female participants had the lowest mean level (9.65 pg/mL), and male participants had the lowest median (9.58 pg/mL). People living in the community between 2 and 4 years old had a lower mean and median (9.03 pg/mL and 9.17 pg/mL). The highest mean was found among those living in the community for over ten years (9.80 pg/mL). Workers who reported having direct contact with pesticides had lower mean and median values (9.62 pg/mL and 9.64 pg/mL).

The dosage of IL-10 according to food intake frequency showed lower mean and median levels among those who consumed game 1–2 times during the week (9.30 pg/mL and 9.03 pg/mL), while the average level was higher among those who consumed it 3–4 times (9.98 pg/mL). The average and median were lower among those who consumed fish and seafood daily (8.66 pg/mL and 8.79 pg/mL, respectively), and the highest average was found among those who consumed them five to six times during the week (10.06 pg/mL). A lower mean was observed among those who claimed to consume fruit daily (9.21 pg/mL), the median was lower among those who consumed them 5–6 times (9.10 pg/mL), and the average was higher among those who claimed not to consume them (11.05 pg/mL). The mean and median were lower among those who consumed vegetables daily (9.02 pg/mL and 9.03 pg/mL), and the highest mean was among those who did not respond (10.96 pg/mL). In this group, a statistically significant difference was observed between the mean levels of IL-10 according to the frequency of consumption of greens and legumes (*p* < 0.04).

## 4. Discussion

### 4.1. Cholinesterase Enzyme Activity

Inhibition of AChE and BChE enzymes is a fundamental parameter for characterizing human environmental exposure to cholinesterase inhibitor pesticides, especially considering that the main pesticides used in large-scale agriculture are neurotoxic [31,32]. In this study, the activity of AChE and BChE in blood was first evaluated in a traditional community exposed to environmental toxins in Pará, in the Northern Region of Brazil.

Considering that basal levels of cholinesterase vary from person to person, the ideal is the pre-exposure determination of each individual for occupational and environmental control of exposure to organophosphates and carbamates. However, this determination is impossible, so commonly used reference values of enzymatic activity obtained from a population not exposed, are BChE = 1.5–3.5 μL/mL, and erythrocyte AChE = 2.6–4.1 μL/mL [28].

In this study, the lowest average levels were the enzyme activity of AChE and BChE identified in children aged 1–11 years (4.89–4.21 μL/mL). It is important to note that this group is considered at risk for pesticide exposure due to its greater susceptibility to harmful effects and even permanent health complications, as they are in the physical and neurological development phase. The great concern with the vulnerability of the developing neurological system is the occurrence of neurotoxicological effects due to sustained exposure to low doses [7]. These data underscore the potential health risks associated with pesticide exposure, particularly in vulnerable populations. They also highlight the need for further research and the implementation of targeted public measures to mitigate these risks.

In Ecuador, Suarez-Lopez et al. [33] studied the effects of anticholinesterase pesticides among children living with flower planting workers, comparing them to a group of non-exposed ones, whose mean acetylcholinesterase value was 3.22 μL/mL. Values below those found among the group of children from the Santo Antônio community suggest that it is not a worrying mean value regarding the activity of the AChE enzyme. Valencia-Quintana et al. [34] did not observe inhibition of cholinesterase activities (AChE and BChE) in 54 agricultural workers in Mexico.

Female participants had mean and median lower AChE (5.35 and 4.74 μL/mL), and mean BChE equal to male participants (4.64 μL/mL), unlike most studies in the literary review of the present study. Our results were similar to the work carried out by Thetkathuek et al. [35] with workers exposed to pesticides in Thailand, which revealed that men had a higher risk of having a reduction in AChE activity than women (1.58 times). In a previous study, Namwong et al. (2011) reported that male workers were at greater risk for cholinergic effects and were more directly exposed to pesticides than female workers during pesticide preparation and spraying. Kapeleka et al. [36] conducted a comparative cross-sectional study of 90 farmers occupationally exposed to pesticides and 61 individuals as a control group. The results of AChE tests showed that exposed farmers had decreased AChE activity compared to the control group. In the same study. AChE levels in women were lower than those of exposed men and their female counterparts in the control group.

Although most workers who reported having direct contact with pesticides are male (96%), it is essential to consider other potential sources of contaminants to which these women may also be exposed [24]. Dendezais are predominantly near or crossing water courses, streams, rivers, and lakes. Therefore, the chemical products used in the plantations are carried by leaching in the region or infiltration into these liquid bodies. Oil palm areas present environmental risks due to possible contamination of water resources, compromising the health of traditional communities in and around these bodies of water. This highlights the importance of considering all potential sources of contaminants for a comprehensive risk assessment to ensure the health and safety of these communities. This is consistent with another result found by this study, which demonstrates that workers who reported not having direct contact with pesticides had lower mean AChE and BChE (5.57 and 4.60 μL/mL) than those with direct contact.

AChE and BChE had lower means among community members for between 2 and 4 years (5.27 μL/mL and 4.15 μL/mL). Statistical significance was observed for mean levels of BChE by the frequency of fish and seafood consumption (*p* < 0.03), with a progressive decrease in BChE activity among those who did not consume this food up to five and six times. In Amazonia, fish consumption is much higher than in other regions of Brazil. Taking water resources into account as possible sources of exposure to pesticides, and the fact that many environmental chemical pollutants are absorbed by the digestive route, together with food from their presence in the food chain. More detailed analyses must be carried out on these foods so that possible associations with human poisoning can be better understood [37].

While the analysis of the blood samples collected showed that, in most cases, there was no significant reduction in the activity of AChE and BChE, these results indicate that laboratory tests are not definitive for diagnosing poisoning by organophosphates or carbamates. It is essential to point out that the effects of pesticides can cause not only enzymatic changes but also functional changes in organs and tissues [38]. Therefore, it is necessary not only for laboratory investigations but also for clinical investigations to close the diagnosis of exposition by organophosphates and carbamates. For the execution of a successful monitoring program, it must be taken into account that some conditions in addition to exposure to pesticides can alter the activity of ChEs, impairing the evaluation and interpretation of the results of the studies. Other factors that can result in misinterpretation of ChE activity levels are improper collection processing transport and, laboratory analysis errors [32]. In addition, responses to epidemiological questionnaires may contain erroneous information that could lead to errors in interpreting cholinesterase activity. This underscores the need for further research and monitoring to fully understand and mitigate the health effects of pesticide exposure

In this study, the lack of significant reduction in AChE and BChE levels may have been because the exposure to pesticides in most individuals was environmental or was not high enough to produce an effect that could have caused considerable enzyme inhibition. A limitation of this study was not knowing when the last period of pesticide application in the palm oil crop was.

### 4.2. Cytokines

#### 4.2.1. Pro-Inflammatory

Cytokines can act on the human body in synergistic or antagonistic forms, constituting a network resulting in a delicate balance determining the body’s homeostasis. This balance shows vulnerabilities to chemicals including pesticides, which may lead to structural changes and function in the immune system, causing severe health problems. Although several studies have investigated the effects of pesticide exposure on the cytokine network, most of these studies examined the effects in vitro, while in vivo studies have focused primarily on model animals [11]. Therefore, for this work, reference values from the Arup Laboratory cytokine panel were used, which used the same cytokine quantification methodology as this research. IFN-γ < 5.00 pg/mL. TFN < 22.00 pg/mL, IL-2 < 1033.00 pg/mL, IL-4 < 5.00 pg/mL, IL-6 < 5.00 pg/mL, and IL-10 < 18.00 pg/mL.

In this study, among the pro-inflammatory cytokines, the highest means of IFN-γ (9.28 pg/mL), TNF (8.97 pg/mL), and IL-6 (35.92 pg/mL) by age group were in those over 60 years old. This may be related to natural factors, such as the constant increase in the basal inflammatory response and aging in general.

Proskocil et al. [15] demonstrated that subacute doses of the organophosphates chlorpyrifos and diazinon increased the production of TNF and IL-6. Increased serum levels of these cytokines are implicated in numerous inflammatory diseases, including those more prevalent in the old, such as Alzheimer’s disease, atherosclerosis, diabetes, and cancer [39].

Regarding gender, the mean levels of IFN-γ (9.35 pg/mL) and IL-6 (23.28 pg/mL) were higher among female participants, with levels above reference values (>5.00 pg/mL) used in this study and far higher than those presented as a risk factor for mortality [40], where they mention that IL-6 levels greater than 0.03 pg/mL were associated with twice the risk of death. Statistical significance was observed between IFN-γ (*p* < 0.02) and IL-6 (*p* < 0.008) levels by sex.

The mean levels of IFN-γ and IL-6 above the reference values in workers who reported having direct contact with pesticides may be related to constant pesticide exposure. Organophosphates induce changes in the morphology of immunocompetent cells, inhibit anti-inflammatory mechanisms, and influence cytokine cascades involved in preventing apoptosis [41]. It is important to note that the wider the exposure to pesticides, the more toxic feeding occurs by ingestion of contaminants, which reach the digestive tract tissues or storage in adipose tissue or the liver [42]. This study showed that the highest mean level of TNF was among those who consumed wild animals 3–4 times a week (10.51 pg/mL) compared to the other frequencies (*p* > 0.001). The average was higher among those who consumed fish and seafood 1–2 times per week (9.01 pg/mL), and the difference from the average levels of TNF was statistically significant (*p* > 0.001).

The IL-2 dosage was observed to be higher among those who claimed to consume fruit once to twice during the week (9.76 pg/mL) (*p* > 0.0003). In a report released by the National Health Surveillance Agency of Brazil (ANVISA) in 2019 through the Program for the Analysis of Pesticide Residues in Food, which tested 4.616 samples of 14 foods. Imidacloprid was the most abundant [43]. It is a neonicotinoid—a nicotine-derived insecticide that can spread to all parts of the plant. It is hazardous because washing or peeling the food is insufficient to remove the pesticide residues inside the plant [43]. Research has shown the need for more studies on pesticide exposure, especially in analyzing the water and food consumed by this community, as demonstrated by the significant relationship between increased levels of pro-inflammatory cytokines and consumption of game, fish, seafood, and fruits.

Pesticides can affect human health. depending on the form and time of exposure and their specific toxicity. The effects can be acute by short-term exposure, that is, hours and for a few days, showing signs of typical intoxication of these products. However. long-term effects through prolonged exposure for more than a year can cause diseases [44].

There was an increase in the levels of IFN-γ cytokines, especially IL-6, in all groups analyzed in this study. IL-6 is a glycoprotein secreted by many types of cells, such as macrophages, monocytes, eosinophils, hepatocytes, and glial cells, and is a potent inducer of TNF-α and IL-1 [45]. It causes fever and activates the hypothalamic-pituitary-adrenal axis. Interleukins are one of the earliest and most essential mediators of induction and control of the synthesis and release of acute-phase proteins by hepatocytes during painful stimuli such as trauma, infection surgery, and burns [46]. It is considered the most relevant marker of the degree of tissue damage during surgical procedures, in which excessive and prolonged increases are associated with more significant postoperative morbidity. It is also related to the vulnerability and erosion of the atherosclerotic plaque [47].

IFN-γ is produced by several cells, such as B lymphocytes. NK cells, antigen-presenting cells, and T lymphocytes. Interferons participate in cell control and replication and are immune response modifiers with antiviral, antiproliferative, and immunomodulatory effects. As this cytokine regulates the immune response, its production can lead to autoimmune diseases [37].

#### 4.2.2. Anti-Inflammatory

The cytokines may have pro- (Th1) or anti-inflammatory (Th2) effects, depending on the microenvironment in which they are located. Anti-inflammatory drugs are a series of immunoregulatory molecules that control the responses of pro-inflammatory cytokines, including IL-10 and IL-4 [48].

In the study, the highest mean levels of IL-4 (10.01 pg/mL) and IL-10 (9.84 pg/mL) were among adults aged 20–59 years compared to other age groups, with IL-4 being above the reference value (<5.00 pg/mL), who demonstrated that carbaryl, a type of carbamate, reduced the weight of the thymus and spleen in rats, in addition to altering the Th1/Th2 balance and increasing the production of IL-4 [49]. Regarding sex, IL-4 showed a higher mean among female participants (10.30 pg/mL). It was observed that people residing in the community between 2 and 4 years had the highest average IL-4 level (10.50 pg/mL), being above the reference value.

The workers who reported direct contact with pesticides had a higher mean level of IL-4 (10.59 pg/mL). However, the average level of IL-10 was higher among those who did not have direct contact with these products. Regarding the consumption of vegetables and legumes, the highest mean was among those who did not respond (10.96 pg/mL). In this group, a significant difference was observed between the average levels of IL-10 (*p* < 0.04). Santos et al. [50] analyzed blood samples from 187 rural workers with breast cancer, Occupationally or not exposed to pesticides, to quantify the levels of the cytokines IL-1β. IL-12, IL-4, IL-17-A, and TNF-α. IL-12, IL-17A, and IL-4 were reduced in women with lymph nodes.

IL-4 levels above the reference value in all groups are noteworthy. They align with the findings of this research about the high levels of IL-6 observed, as it has a direct structural relationship with IL-4. acting as an antagonist in the inflammatory process elicited by IL-6 [51]. IL-4 is a glycoprotein with anti-inflammatory properties produced by CD4-T-lymphocytes, mast cells, eosinophils, and basophils. It acts on T and B lymphocytes, natural killers, masts, synovial, and cells [51,52]. It induces B-lymphocyte differentiation to produce IgG and IgE—important immunoglobulins in allergic and antihelminthic responses. It acts on activated macrophages, reducing the effects of the cytokines IL-1, TNFα, IL-6, and IL-8, and inhibits the production of oxygen-free radicals. IL-4 has therapeutic potential in many clinical situations, such as psoriasis, tartrate, lymphoma, and asthma, but their values must be further investigated [52].

## 5. Conclusions

The analysis of the blood samples collected showed that, in most cases, there was no significant reduction in the activity of AChE and BChE compared to other studies. An increase in the levels of IFN-γ cytokines, especially IL-6, can also be seen in all groups analyzed in this study. IL-4 levels above the reference value in all groups are noteworthy and are in line with the findings of this study about high levels of IL-6. Although the average cholinesterase values were insignificant. IL-6 and IL-4 values were above the reference values and those in other studies, demonstrating the importance of performing measurements of other biomarkers by the traditionally used ones to assess pesticide exposure, such as AChE enzyme activity. The study results have significant implications for public health, underlining the potential benefits of future comparisons in epidemiological studies of human exposure to pesticides in populations in Brazil and other countries.

The quilombolas, who belong to vulnerable populations in the Amazon, are descendants of enslaved people and live in a community very close to palm oil monocultures that use large amounts of pesticides on their plantations. In addition to being close to the plantations, these quilombolas have lived in the community for more than ten years, with continuous environmental exposure. To improve the community’s living and health conditions, it is also necessary to define actions such as intensifying specific public policies for generating employment and income independent of palm oil activity, expanding health policies aimed at Primary Health Care and encouraging the development of agroecological practices among families in the community evaluated in this study. It was the first study to address exposure to biomarkers, mainly cytokines, in traditional Amazonian communities, such as quilombolas.

## Figures and Tables

**Table 1:** Demographic data of participants in the Santo Antônio/Concórdia do Pará Quilombola Community, Brazil.

Variables	Extract	Frequency	
		*n* (188)	%
Age Group	1–11	24	12.8
	12–19	32	17.0
	20–59	106	56.4
	≥60	26	13.8
Sex	Female	87	46.3
	Male	101	53.7
Time dwelling in the community	2 to 4 years	15	8.0
	5 to 10 years	15	8.0
	>10 years	158	84.0
Works directly with pesticides	Yes	25	13.3
	No	163	86.7
BMC by age group	1 to 5 years		
	Thinness	2	15.4
	Eutrophy	9	69.2
	Overweight Risk	2	15.4
	6 to 19 years		
	Eutrophy	39	90.7
	Overweight	3	7.0
	Obesity	1	2.3
	20 to 59 years old		
	Low weight	1	0.9
	Eutrophy	36	34.0
	Overweight	49	46.2
	Obesity	20	18.9
	≥60		
	Low weight	3	11.5
	Eutrophy	10	38.5
	Overweight	13	50.0

**Table 2:** Statistical analysis of acetylcholinesterase and butyrylcholinesterase enzyme activity.

Variable	AChE (UI/mL)				BChE (UI/mL)			
	M ± DP	Md	Min–Max	*p*	M ± DP	Md	Min–Max	*p*
**Age Group (years)**								
1–11	4.89 ± 2.77	4.47	1.00–12.20	0.73	4.21 ± 1.97	3.82	1.84–7.53	0.55
12–19	5.85 ± 3.27	5.71	1.04–11.79		4.59 ± 2.36	4.53	0.93–10.16	
20–59	5.86 ± 3.61	5.09	0.07–14.26		4.60 ± 1.86	4.55	1.11–8.95	
≥60	5.56 ± 2.95	5.32	0.04–11.18		5.24 ± 2.52	4.84	2.05–10.18	
**Sex**								
Female	5.35 ± 3.13	4.74	0.04–11.79	0.31	4.64 ± 1.95	4.56	1.78–10.18	0.94
Male	5.99 ± 3.54	5.23	0.07–14.26		4.64 ± 2.17	4.54	0.93–10.16	
**Living time in the community (years)**								
2–4	5.27 ± 3.33	3.82	0.71–12.20	0.84	4.15 ± 1.92	3.88	2.01–7.53	0.57
5–10	5.40 ± 3.12	4.70	1.00–11.79		4.86 ± 2.07	5.75	1.78–7.90	
>10	5.76 ± 3.40	5.19	0.04–14.26		4.66 ± 0.93	4.54	0.93–10.18	
**Works directly with pesticides**								
Yes	6.47 ± 4.44	5.15	0.47–14.26	0.57	4.89 ± 2.14	5.26	1.30–9.49	0.46
No	5.57 ± 3.17	5.10	0.04–13.40		4.60 ± 2.06	4.54	0.93–10.18	
**Food Frequency**								
Hunting								
Does not consume	5.66 ± 3.48	5.01	0.04–14.26	0.81	4.63 ± 2.00	4.55	0.93–10.16	0.61
1 to 2/week	5.79 ± 2.83	5.31	1.50–12.48		4.56 ± 2.21	4.36	1.25–10.18	
3 to 4/week	6.44 ± 4.17	5.62	2.74–10.96		6.09 ± 3.55	6.09	2.39–9.49	
Fish and seafood								
Does not consume	5.58 ± 4.21	4.46	1.09–11.83	0.09	4.93 ± 2.05	4.57	2.32–8.67	0.03 **
1 to 2/week	5.17 ± 3.08	4.48	0.04–13.15		4.63 ± 2.10	4.56	0.93–10.16	
3 to 4/week	6.61 ± 3.81	6.77	0.07–13.40		4.81 ± 1.88	4.68	1.18–9.48	
5 to 6/week	6.32 ± 2.81	5.34	3.20–11.77		3.21 ± 1.30	2.91	1.62–6.87	
Daily	7.31 ± 3.99	6.12	0.71–14.26		5.84 ± 2.43	5.93	2.46–10.18	
Fruit								
Does not consume	5.77 ± 7.48	5.77	0.47–11.06	0.41	5.62 ± 1.53	5.62	4.54–6.71	0.29
1 to 2/week	5.35 ± 3.32	4.51	0.04–12.20		4.57 ± 2.11	4.56	0.93–10.16	
3 to 4/week	5.37 ± 3.34	4.66	0.07–13.15		4.65 ± 1.87	4.53	1.18–8.95	
5 to 6/week	6.89 ± 3.52	6.66	2.18–14.26		4.08 ± 1.90	3.61	1.11–7.90	
Daily	5.79 ± 3.18	5.70	0.71–13.40		5.08 ± 2.29	5.11	1.25–10.18	
They did not answer	9.03 ± *	9.03	9.03–9.03		2.19 ± *	2.19	2.19–2.19	
Vegetables								
Does not consume	4.96 ± 3.03	3.73	1.00–11.06	0.71	5.10 ± 2.55	4.63	1.25–9.17	0.31
1 to 2/week	5.61 ± 3.33	4.74	0.04–14.26		4.45 ± 1.93	4.54	0.93–10.16	
3 to 4/week	5.60 ± 3.56	4.35	0.07–12.18		4.73 ± 1.93	4.58	1.18–8.95	
5 to 6/week	6.39 ± 2.62	6.09	2.46–10.76		4.37 ± 2.31	3.63	2.00–8.53	
Daily	6.00 ± 3.82	5.32	1.00–13.40		5.14 ± 2.27	5.10	1.62–10.18	
They did not answer	7.50 ± 2.16	7.50	5.98–9.03		2.26 ± 0.09	2.26	2.19–2.32	
**BMC by Age Group**								
1 to 5 years								
Thinness	6.42 ± 3.68	6.42	3.82–9.03	0.46	4.52 ± 3.29	4.52	2.19–6.85	0.89
Eutrophy	4.46 ± 3.43	2.69	1.65–12.20		4.40 ± 2.03	4.38	2.28–7.53	
Overweight Risk	3.81 ± 3.06	3.81	1.64–5.98		3.76 ± 2.03	3.76	2.32–5.20	
6 to 19 years								
Eutrophy	5.75 ± 3.06	5.80	1.00–13.40	0.30	4.37 ± 2.27	4.22	0.93–10.16	0.09
Overweight	3.79 ± 2.42	4.70	1.04–5.62		6.27 ± 0.12	6.30	6.14–6.39	
Obesity	8.20 ± *	8.20	8.20–8.20		2.46 ± *	2.46	2.46–2.46	
20 to 59 years old								
Low weight	7.50 ± *	7.50	7.50–7.50	0.38	2.26 ± *	2.26	2.26–2.26	0.17
Eutrophy	6.54 ± 3.79	6.16	0.07–14.26		4.21 ± 1.78	4.00	1.11–8.90	
Overweight	5.72 ± 3.55	4.48	0.10–13.15		4.95 ± 2.05	4.97	1.62–8.95	
Obesity	4.90 ± 3.42	4.37	0.71–12.48		4.58 ± 1.41	4.74	1.30–6.87	
≥60								
Low weight	5.63 ± 4.71	6.32	0.60–9.96	0.22	4.43 ± 2.32	4.56	2.05–6.69	0.82
Eutrophy	6.70 ± 2.11	6.03	3.79–9.44		5.34 ± 2.75	5.58	2.14–10.18	
Overweight	4.67 ± 3.04	4.35	0.04–11.18		5.36 ± 2.55	4.42	2.28–9.88	

* 1 participan only/not applicable. ** Chi-square or Kruskal–Wallis test.

**Table 3:** Statistical analysis of cytokines.

Variable	IFN-γ (pg/mL)			TNF (pg/mL)			IL-2 (pg/mL)			IL-4 (pg/mL)			IL-6 (pg/mL)			IL-10(pg/mL)		
	M ± DP	Md	*p*	M ± DP	Md	*p*	M ± DP	Md	*p*	M ± DP	Md	*p*	M ± DP	Md	*p*	M ± DP	Md	*p*
Age Group (years)																		
1–11	9.20 ± 2.79	9.34	0.27	8.28 ± 1.74	8.05	0.23	9.18 ± 1.18	9.10	0.49	9.78 ± 2.21	9.76	0.84	18.65 ± 10.51	14.72	0.55	9.28 ± 1.42	9.33	0.67
12–19	8.74 ± 1.98	8.99		8.97 ± 1.92	8.84		9.61 ± 1.32	9.36		9.97 ± 2.92	9.30		17.85 ± 12.94	12.91		9.69 ± 1.40	9.61	
20–59	8.78 ± 2.59	8.91		8.89 ± 1.71	8.95		9.49 ± 1.34	9.36		10.01 ± 3.03	9.64		21.06 ± 45.11	13.67		9.84 ± 1.82	9.79	
≥60	9.28 ± 2.39	9.93		8.73 ± 1.52	8.34		9.34 ± 1.25	9.46		9.33 ± 1.94	9.11		35.92 ± 100.01	14.07		9.53 ± 1.59	9.39	
Sex																		
Female	9.35 ± 2.65	9.39	0.02 ***	8.72 ± 1.96	8.48	0.30	9.38 ± 1.36	9.18	0.56	10.30 ± 2.81	9.64	0.06	23.28 ± 49.08	14.48	0.008 ***	9.65 ± 1.47	9.76	0.79
Male	8.51 ± 2.29	8.57		8.88 ± 1.50	8.91		9.51 ± 1.25	9.48		9.52 ± 2.71	9.27		21.39 ± 51.96	13.34		9.74 ± 1.84	9.58	
Living time in the community (years)																		
2–4	9.25 ± 2.83	9.45	0.24	8.21 ± 1.50	7.71	0.06	8.84 ± 1.17	8.43	0.06	10.50 ± 2.25	10.51	0.41	19.28 ± 12.04	14.88	0.24	9.03 ± 1.46	9.17	0.27
5–10	10.21 ± 2.96	9.38		8.54 ± 2.51	8.56		9.20 ± 0.97	9.12		10.23 ± 3.92	9.27		19.04 ± 8.05	15.03		9.34 ± 1.34	9.29	
>10	8.74 ± 3.38	8.99		8.89 ± 1.66	8.84		9.53 ± 1.33	9.42		9.79 ± 2.70	9.53		22.85 ± 54.95	13.67		9.80 ± 1.71	9.76	
Works directly with pesticides																		
Yes	8.03 ± 2.10	8.44	0.04 ***	82.90 ± 1.74	8.74	0.85	9.45 ± 1.19	9.45	0.95	10.59 ± 3.49	10.08	0.33	17.11 ± 10.93	13.78	0.84	9.62 ± 1.51	9.64	0.42
Food Frequency																		
Hunting																		
Does not consume	8.75 ± 2.61	8.92	0.05	8.96 ± 1.55	8.90	0.001 **	9.45 ± 1.23	9.38	0.77	9.78 ± 2.73	9.55	0.75	20.04 ± 43.18	13.70	0.14	9.79 ± 1.72	9.79	0.30
1 to 2/ week	9.34 ± 1.84	9.68		7.98 ± 2.00	7.80		9.48 ± 1.58	9.18		10.21 ± 2.84	9.42		32.65 ± 75.67	15.33		9.30 ± 1.45	9.03	
3 to 4/ week	10.89 ± 1.98	10.29		10.51 ± 3.59	9.07		9.08 ± 1.57	8.47		11.16 ± 4.94	8.99		12.40 ± 1.85	12.77		9.98 ± 1.86	9.11	
Fish and seafood																		
Does not consume	10.99 ± 3.66	10.63	0.12	8.82 ± 0.65	8.91	0.001 **	9.44 ± 0.91	9.88	0.16	10.36 ± 1.69	10.49	0.57	19.21 ± 18.11	12.29	0.07	9.90 ± 1.15	10.08	0.27
1 to 2/ week	8.63 ± 2.32	8.94		9.01 ± 1.78	8.95		9.43 ± 1.21	9.24		9.68 ± 2.77	9.27		20.45 ± 47.97	13.67		9.70 ± 1.72	9.68	
3 to 4/ week	9.00 ± 2.98	9.04		9.00 ± 1.61	9.07		9.51 ± 1.40	9.38		10.22 ± 3.25	9.65		18.35 ± 16.16	13.73		9.79 ± 1.44	9.30	
5 to 6/ week	10.03 ± 1.76	10.03		7.52 ± 1.12	7.57		10.13 ± 1.52	10.18		10.08 ± 2.26	9.76		53.05 ± 118.44	21.55		10.06 ± 2.27	9.97	
Daily	8.75 ± 1.50	9.04		7.45 ± 1.50	7.86		8.55 ± 1.43	8.90		10.25 ± 1.94	10.33		18.27 ± 12.56	15.74		8.66 ± 1.25	8.79	
Fruit																		
Does not consume	8.74 ± 2.41	8.74	0.68	9.63 ± 0.40	9.63	0.0003 **	9.47 ± 1.28	9.47	0.0003 **	9.54 ± 1.36	9.54	0.77	13.29 ± 0.62	13.29	0.37	11.05 ± 0.84	11.05	0.07
1 to 2/ week	8.39 ± 2.44	8.38		9.48 ± 1.66	9.64		9.76 ± 1.02	9.74		9.77 ± 2.72	9.53		16.05 ± 10.01	13.96		9.99 ± 1.73	9.87	
3 to 4/ week	9.12 ± 2.27	9.13		8.73 ± 1.72	8.51		9.64 ± 1.63	9.21		10.32 ± 2.91	9.75		29.94 ± 771.93	13.92		9.74 ± 1.99	9.76	
5 to 6/ week	9.29 ± 2.68	9.12		8.27 ± 1.64	8.36		9.56 ± 1.34	9.34		9.96 ± 3.67	9.87		33.42 ± 87.66	14.04		9.56 ± 1.54	9.10	
Daily	9.12 ± 2.17	9.35		8.20 ± 1.63	8.13		8.71 ± 0.94	8.78		9.45 ± 2.07	9.27		15.84 ± 10.15	12.85		9.21 ± 1.14	9.30	
They did not answer	9.66 ± *	9.66		7.69 ± *	7.69		8.95 ± *	8.95		11.18 ± *	11.18		24.80 ± *	24.80		11.06 ± *	11.06	
Vegetables																		
Does not consume	8.51 ± 1.55	8.80	0.37	8.48 ± 1.02	8.80	0.02 **	9.18 ± 1.04	8.84	0.13	8.77 ± 1.40	9.27	0.12	15.63 ± 5.88	13.30	0.14	10.21 ± 2.85	9.49	0.04 **
1 to 2/ week	8.67 ± 2.52	8.56		9.21 ± 1.74	9.29		9.52 ± 1.15	9.50		9.70 ± 2.79	9.51		15.82 ± 9.46	13.67		9.91 ± 1.33	9.87	
3 to 4/ week	9.21 ± 2.53	9.39		8.69 ± 2.09	8.54		9.74 ± 1.66	9.48		10.53 ± 3.04	9.93		30.49 ± 67.93	14.10		9.56 ± 1.93	9.56	
5 to 6/ week	9.25 ± 1.88	9.49		8.47 ± 1.20	8.34		9.71 ± 1.26	9.55		10.33 ± 2.21	11.09		52.82 ± 136.22	13.43		9.47 ± 1.68	9.33	
Daily	8.78 ± 2.69	9.23		8.16 ± 1.38	8.16		8.87 ± 1.07	9.04		9.63 ± 3.02	9.19		15.32 ± 9.46	13.30		9.02 ± 1.23	9.03	
They did not answer	13.23 ± 5.05	13.23		7.91 ± 0.31	7.91		8.43 ± 0.73	8.43		11.54 ± 0.50	11.54		40.39 ± 22.05	40.39		10.96 ± 0.13	10.96	
BMC by Age Group																		
1–5 years																		
Thinness	9.42 ± 0.33	9.42	0.83	7.91 ± 0.31	8.32	0.58	8.64 ± 0.43	8.64	0.96	10.84 ± 0.47	10.84	0.46	17.97 ± 9.65	17.97	0.49	10.73 ± 0.45	10.73	0.12
Eutrophy	8.79 ± 2.18	8.94	0.36	8.53 ± 1.70	8.48	0.21	9.57 ± 1.28	9.38	0.71	9.77 ± 2.37	9.55	0.84	17.57 ± 12.10	13.00	0.71	9.55 ± 1.41	9.58	
Overweight	10.14 ± 0.70	10.29		11.62 ± 3.52	10.16		9.85 ± 2.00	8.94		11.69 ± 6.37	8.35		18.18 ± 10.66	13.82		10.29 ± 1.15	10.35	
Obesity	9.60 ± *	9.60		8.45 ± *	8.45		8.88 ± *	8.88		8.40 ± *	8.40		18.67 ± *	18.67		7.77 ± *	7.77	
20–59 years																		
Low weight	13.18 ± *	13.18	0.27	8.96 ± *	8.96	0.97	9.56 ± *	9.56	0.59	10.16 ± *	10.16	0.25	23.05 ± *	23.05	0.17	8.65 ± *	8.65	0.37
Eutrophy	9.04 ± 2.37	9.34		8.96 ± 1.80	9.02		9.43 ± 1.58	9.42		9.31 ± 2.80	8.57		17.04 ± 15.22	13.30		9.72 ± 2.45	9.38	
Overweight	8.66 ± 2.78	8.55		8.77 ± 1.70	8.95		9.42 ± 1.24	9.17		10.56 ± 3.38	10.08		25.72 ± 64.87	13.67		9.94 ± 1.50	9.87	
Obesity	8.38 ± 2.45	8.74		9.07 ± 1.68	9.02		9.79 ± 1.17	9.70		9.93 ± 2.36	9.27		16.79 ± 8.88	15.02		9.86 ± 1.21	9.92	
Low weight	9.02 ± 3.37	7.62	0.42	9.39 ± 0.92	9.85	0.40	9.40 ± 1.48	9.61	0.65	9.27 ± 2.16	9.04	0.06	13.09 ± 2.54	12.78	0.04 **	8.52 ± 1.36	8.10	0.10

* 1 participant only/not applicable. ** Chi-square or Kruskal–Wallis test. *** Mann–Whitney test.

## Data Availability

The original contributions presented in the study are included in the article.
